# Addressing communication dynamics in traditional medicine use disclosure to physicians

**DOI:** 10.4102/phcfm.v18i1.5177

**Published:** 2026-01-27

**Authors:** Lindiwe Gumede

**Affiliations:** 1 Department of Medical Imaging and Radiation Sciences, Faculty of Health Sciences, University of Johannesburg, Johannesburg, South Africa

**Keywords:** traditional medicine, allopathic medicine, disclosure, communication privacy management theory, cultural safety, South Africa

## Abstract

**Background:**

Although traditional medicine (TM) has historically been marginalised within the allopathic medicine (AM) systems and policy frameworks, it remains a core component of healthcare-seeking behaviour among South Africans. However, communication regarding TM use between patients and physicians is often inhibited by cultural stigma, trust deficits and isolated systems.

**Aim:**

This study explored physicians’ perceptions of communication dynamics influencing TM disclosure, guided by communication privacy management (CPM) theory.

**Setting:**

The study setting was four district hospitals with outpatient departments in Gauteng province.

**Methods:**

This qualitative descriptive study collected data from 14 physicians through semi-structured interviews. The findings were inductively analysed using open and axial coding, with CPM theory serving as a sensitising framework.

**Results:**

Four interrelated themes aligned with CPM theory’s core concepts: (1) disclosure practices, (2) facilitation of collaboration, (3) managing disclosed information and (4) challenges in non-disclosure. Physicians reported that patients regulate TM disclosure according to trust, perceived judgement and cultural norms. Respectful, non-judgemental communication fostered openness, whereas dismissive attitudes reinforced secrecy.

**Conclusion:**

Communication privacy management theory provided a valuable lens for understanding physicians’ perceptions of privacy management within South Africa’s dual healthcare context. Disclosure of TM is a relational and culturally mediated process shaped by social trust and institutional dynamics.

**Contribution:**

The study contributes theoretically by extending CPM theory to a multicultural and interprofessional setting; methodologically by illustrating its use as a sensitising framework for analysing healthcare communication; and practically by identifying strategies such as cultural humility training, structured disclosure frameworks and collaboration between TM and AM practitioners that can strengthen transparency and patient-centred care.

## Introduction

South Africa’s healthcare landscape is characterised by multiple coexisting health systems – allopathic, traditional, spiritual and alternative – reflecting the country’s cultural diversity and plural health-seeking patterns.^1,2,3,4^ Many South Africans use traditional medicine (TM) not only as a complement to allopathic medicine (AM) but also as an autonomous, trusted system of care that aligns with their cultural values, worldviews and community networks.^5,6,7^ Many patients engage with multiple systems concurrently, combining AM, TM and spiritual approaches to care, drawing from whichever offers what has been described in medical anthropology as ‘helpful help’.^8,9^ This is regarded as the search for care perceived to be effective or meaningful. Rather than a binary choice between TM and AM, this dynamic pluralism illustrates how people integrate multiple treatment options to meet physical, emotional and spiritual needs.

However, despite this plural reality, communication between patients and physicians about TM use remains limited, inconsistent and often marked by tension. Many patients avoid disclosing TM because of fear of stigma,^10^ and perceived lack of trust,^11^ while physicians may feel uncertain about how to manage such disclosures within AM frameworks. This lack of open communication can lead to clinical risks such as adverse interactions between TM and prescribed medicines, fragmented care and missed opportunities for culturally responsive practice.^12^

While prior research in South Africa and beyond has explored patterns of TM use and models for integrating biomedical and traditional systems,^13,14^ much less attention has been paid to the communication dynamics that shape how TM use is discussed within AM consultations. Studies^15,16,17^ tend to frame integration as a policy or structural issue, overlooking the interpersonal and relational negotiations that occur when patients decide whether, how and to whom to disclose TM use. This gap limits understanding of the everyday communicative processes through which plural healthcare practices are managed in clinical settings.

This study addresses that gap by exploring how physicians in South Africa navigate communication about TM use disclosure through the lens of communication privacy management (CPM) theory. Communication privacy management theory offers a nuanced framework for analysing how individuals manage private health information, defining boundaries, establishing disclosure rules, coordinating shared information and responding to turbulence when boundaries are breached.^18^ By applying CPM theory within the South African context, this study seeks to illuminate how cultural legitimacy, professional authority and relational trust intersect to shape disclosure practices. Ultimately, it contributes to improving communication strategies that foster patient safety, mutual respect and culturally competent, integrative care.

### Conceptual framework: Communication privacy management theory

Communication privacy management theory has become a prominent framework in health communication research, offering insight into how individuals regulate the disclosure of private health information.^19^ Originally developed by Sandra Petronio, CPM theory has been applied in diverse healthcare contexts and patient–provider interactions.^20,21^ These applications demonstrate CPM theory’s flexibility in explaining how patients and practitioners negotiate privacy boundaries, trust and ownership of sensitive information in clinical encounters.

Samens^22^ highlights that disclosure of health information plays a central role in shaping relationships and decision-making within care contexts, while Bylund et al.^23^ identify CPM theory as a guiding framework for understanding interpersonal communication in healthcare. In these studies, private health information is viewed as a form of owned property that individuals seek to control through culturally and relationally informed privacy rules.

Communication privacy management theory comprises four interrelated components: ownership, privacy rules, boundary coordination and boundary turbulence.^18^ ‘Ownership’ refers to the perception that individuals are the primary custodians of their private information, such as their use of TM. Privacy rules govern the conditions under which this information is shared, influenced by factors like trust, risk and perceived benefit. When private information is disclosed, boundary coordination occurs, requiring co-management of the shared information between patients and practitioners. Boundary turbulence arises when these boundaries are violated, misunderstood or poorly managed, often resulting in conflict or loss of trust.

Although CPM theory originated in interpersonal communication research, it has already been applied in South African disclosure and health communication contexts, including studies on HIV-serodiscordant couples^24^ and university theses examining the disclosure of TM use^25^ and mental health conditions.^26^ These local applications demonstrate CPM theory’s analytical value for understanding how individuals manage sensitive information, negotiate trust and respond to boundary turbulence in plural healthcare settings.

Applying CPM theory to the context of this study advances the use of communication theory in African healthcare research. It explores how physicians negotiate boundary coordination and respond to turbulence arising from TM-related disclosure processes that have critical implications for culturally responsive, patient-centred communication and integrative care.

## Research methods and design

### Study design

This study employed a qualitative descriptive design^27^ to explore the communication dynamics surrounding TM use disclosure from physicians’ perspectives. Although this was not a grounded theory study, certain analytic techniques traditionally associated with grounded theory, specifically open and axial coding, were adopted only as organisational tools to support inductive theme development.^28^ Within a qualitative descriptive design, CPM theory was used as a sensitising framework to guide interpretation rather than as a predetermined coding framework.The intention was not to build new theory but to structure and interpret the data systematically. The COnsolidated criteria for REporting Qualitative (COREQ) research guide the reporting of the study (Online Appendix 1).

### Setting and recruitment

The study was conducted at four district hospitals in Gauteng province, each providing inpatient and outpatient services. These hospitals deliver outpatient healthcare, disease prevention and district-level clinical support. Purposive sampling was used to select participants whose characteristics aligned with the study aims.^29^

Family physicians were chosen because they are primarily responsible for comprehensive patient assessment, diagnosis and care coordination within district hospitals. They are often the first point of contact for patients, manage referrals to specialised services and handle cases where patients disclose or choose not to disclose their TM use. Their training in holistic, patient-centred care and frequent engagement in communication-intensive consultations make them ideally positioned to provide insightful perspectives on disclosure dynamics between patients and allopathic practitioners. While nurses also interact closely with patients, family physicians’ broader oversight of care and central role in decision-making align most directly with this study’s focus on disclosure management as well as TM and AM system collaboration.

The physicians at these hospitals, therefore, offered ideal settings for capturing diverse and heterogeneous perspectives on TM disclosure. Participants were approached personally in their departments and did not know the researcher (L.G.). Each received an information letter explaining the study, along with contact details, enabling informed and voluntary participation. The researcher is a female senior lecturer in Medical Imaging and Radiation Sciences with more than 15 years of clinical experience as a diagnostic radiographer and 6 years of academic experience. Her professional background and involvement in qualitative health sciences research facilitated rapport building with participants within a healthcare context.

### Interviews

Fourteen physicians were interviewed between November 2021 and July 2022. Semi-structured, face-to-face interviews were conducted on hospital premises in areas chosen by participants, each lasting 30 min – 45 min. An audio recorder was used with participants’ permission. The interview was initiated by the collection of demographics to contextualise physicians’ perspectives on TM disclosure within South Africa’s culturally diverse healthcare system. All demographic data was justified in accordance with the *Protection of Personal Information Act* (POPIA, Act No. 4 of 2013).^30^ A pre-tested interview guide (Online Appendix 2) was used, and probing questions encouraged depth and clarity. The researcher kept a reflexive diary documenting personal reflections, assumptions and potential biases, as well as field notes to ensure contextual accuracy.

### Data analysis and management

Audio-recorded interviews were transcribed verbatim and analysed inductively using open and axial coding techniques adapted from grounded-theory procedures.^31^ Analysis followed the principles of qualitative description^27,32^ to identify and organise emerging themes rather than to develop new theoretical constructs. Transcripts were returned to participants for verification of accuracy. Data were managed and analysed using data analysis software (Atlas.ti),^33^ allowing for the creation of projects, grouping of transcripts, quotations, code application and visual network views for final analysis (Online Appendix 3). Supervisors independently reviewed a subset of transcripts using the same codebook to ensure consistency and dependability. Regular debriefing sessions with supervisors supported reflexivity and confirmability of findings. Data collection and analysis occurred concurrently until thematic saturation was reached after the twelfth interview; the remaining two interviews confirmed saturation.

### Trustworthiness

Rigour was maintained through strategies addressing the four pillars of trustworthiness.^34^ Credibility was enhanced through prolonged engagement with participants, member checking of transcripts and peer debriefing with supervisors. Dependability was ensured by maintaining an audit trail that documented all methodological and analytical decisions throughout the research process. Confirmability was supported through reflexive journalling to identify and bracket potential researcher bias, complemented by independent verification of the coding process by supervisors. Transferability was promoted by providing detailed descriptions of the research context, participant characteristics and study procedures, enabling readers to determine the applicability of the findings to similar settings.

### Ethical considerations

Ethical clearance for the study was obtained from the Institutional Research Ethics Committee of the Durban University of Technology (IREC 016/21). Permission to conduct the research was granted by the relevant hospital authorities. Participation was voluntary, and each physician provided written informed consent prior to interviews. To maintain confidentiality, no identifying information was recorded; transcripts were anonymised using participant codes (e.g., P1–P14). Audio files and transcripts were stored in password-protected digital folders accessible only to the researcher and supervisors. Participants were reminded of their right to withdraw at any point without penalty. The study adhered to the ethical principles of the Declaration of Helsinki (1975, revised 2013).^35^

## Results

### Sample

The sample reflected the demographic composition of physicians working in the selected district hospitals, rather than deliberate inclusion or exclusion based on ethnicity. Table 1 illustrates the characteristics of participants, indicating employment experience spanning from 3 years to 36 years. Most participants had more than 10 years of employment experience as physicians. Their ages varied from 28 years to 68 years. Only three participants were below the age of 40 years. All participants self-identified within a male-female binary. However, gender identity was recorded based on self-report rather than predetermined categories. Most of the participants (*n* = 13) held the basic qualification, the Bachelor of Medicine and Bachelor of Surgery (MBChB), while one participant had a Master’s in Family Medicine.

**TABLE 1:** Participants’ characteristics (*N* = 14).

Characteristics	Physicians	
	*n*	%
**Gender**		
Male physician	7	50
Female physician	7	50
**Age (years)**		
28–40	3	21
41–50	6	43
51–68	5	36
**Race**		
Black people	13	93
Indian people	1	7
**Qualification**		
Undergraduate	13	93
Postgraduate	1	7
**Work experience (years)**		
3–5	2	14
6–10	2	14
11–15	4	29
16–20	3	21
21–30	1	7
30+	2	14

*Source:* Gumede, L., 2022, ‘Guidelines for disclosure of traditional medicine use to allopathic medicine practitioners by patients who use both traditional medicine and allopathic medicine at selected hospitals in Gauteng, South Africa’, PhD thesis, Durban University of Technology

### Findings summary

The inductive coding process, guided by grounded-theory principles, yielded four main themes and 11 categories (Table 2) that closely align with the key components of CPM theory. These categories serve as overarching themes reflecting participants’ experiences with privacy management. Each theme represents one of the four CPM theory components – privacy boundaries, disclosure rules, boundary coordination and boundary turbulence – and illustrates how physicians perceive patients’ management of TM use information during clinical interactions. Subcategories were developed through open and axial coding to capture specific patterns within the data.

**TABLE 2:** Study categories.

CPM theory core principle	Theme	Categories	Illustrative quote
Privacy boundaries	Disclosure practices	Barriers to disclosure. Factors encouraging disclosure	‘… Perhaps they are afraid that health care providers will not understand why they are using it [*TM*].’ (P5, 42-year-old male, Jan 2022)
Disclosure rules	Facilitators of collaboration	Patient-centric privacy norms Practitioner sensitivity and approach Managing hesitation	‘… So, it’s best to check our attitude, how we address it, how we ask questions, and how we politely address the treatment that we are going to give the patient. (P12, 60-year-old female, Jan 2022)
Boundary coordination	Managing disclosed information	Responses to disclosure Collaboration with traditional health practitioners Integration challenges	‘… So, I think it’s more like health education, training and getting the traditional healers involved. As they are because they work for some patients as the first point of contact with the actual treatment.’ (P6, 40-year-old female, Jan 2022)
Boundary turbulence	Challenges in non-disclosure	Impacts of non-disclosure Conflict resolution Emotional and professional strain	‘That’s a difficult one! Because on this one as a doctor, you cannot do anything, because if the patient still believes that this kind of medicine [*TM*] helps them, you cannot force them to stop …’ (P10, 61-year-old female, Jan 2022)

*Source:* Adapted from Gumede, L., 2022, ‘Guidelines for disclosure of traditional medicine use to allopathic medicine practitioners by patients who use both traditional medicine and allopathic medicine at selected hospitals in Gauteng, South Africa’, PhD thesis, Durban University of Technology

CPM, communication privacy management; THP, traditional health practitioner.

### Privacy boundaries: Disclosure practices

This theme corresponds with the CPM theory component of privacy boundaries, which explains how individuals manage ownership of their private information. Physicians reported that patients exercise ownership of TM-related information, choosing when and how to disclose based on perceived safety, trust and expected reactions from physicians. Participants acknowledged that stigma, fear of judgement and breaches of confidentiality discourage disclosure.

#### Barriers to disclosure

While most participants identified stigma as a major barrier, they recognise that fear of judgement or perceived conflict between TM and AM also influenced patients’ silence. P13 explained:

‘The other one would be stigma, especially from healthcare workers, because of different religious and healthcare practices. As a result, you may find that among us healthcare workers, others are Christians who do not believe in traditional medicines, so patients are reluctant to disclose because of the stigma.’ (P13, 28-year-old male, July 2022)

Similarly, P5 noted:

‘I think the most common reason patients do not disclose their use of traditional medicine is perhaps that they are afraid that healthcare providers will not understand why they are using it [*referring to TM*], as well as the stigma that most people do not want to disclose that they are using traditional medicine or herbs.’ (P5, 42-year-old male, Jan 2022)

These accounts illustrate how patients establish and protect privacy boundaries by withholding TM use when anticipating negative reactions. P6 added further context:

‘There is still a significant gap between traditional and Western medicine. Traditional medicine is still undervalued and regarded as far inferior by Western medicine practitioners.’ (P6, 40-year-old female, Jan 2022)

This reflects how broader professional attitudes can reinforce patients’ protective boundaries around TM information.

#### Factors encouraging disclosure

Conversely, participants described how specific physician behaviours could lower these boundaries. P9 explained:

‘We should talk properly. Patients have the right to make their own decisions and to know as well. The more information we provide, the clearer their decisions will be.’ (P9, 51-year-old male, Jan 2022)

P4 elaborated that disclosure is situational:

‘Disclosure also depended on the circumstance during a consultation … a desperate patient will reveal everything [referring to all medication they are taking]. If they use both [*TM and AM*] simultaneously, they can even seek counsel.’ (P4, 58-year-old female, Jan 2022)

These reflections show that patients may temporarily relax their privacy boundaries when emotional need or trust outweighs fear of judgement.

### Disclosure rules: Facilitators of collaboration

Aligned with the CPM theory concept of privacy and disclosure rules, this theme explores how patients’ decisions to share information are shaped by perceived risk, cultural expectations and relational trust. Physicians noted that disclosure depends on the patient’s belief in confidentiality and on physicians’ sensitivity to cultural practices.

#### Patient-centric privacy norms

Participants emphasised that patients’ privacy rules are culturally embedded. P12 highlighted the importance of respectful inquiry:

‘If we want the patient to disclose, we should examine our attitude, how we address it, how we ask questions, and how we politely address the treatment that we will give the patient.’ (P12, 60-year-old female, Jan 2022)

This suggests that empathetic communication can modify privacy rules, making disclosure permissible.

#### Practitioner sensitivity and approach

Many participants described how their tone and phrasing influenced patients’ willingness to share. P12 explained:

‘The way the patient should be approached, how the doctor asks the patient questions, how the doctor opens up to the patient, and how the patient opens up to you, that, I believe, is how the patient will reveal that they are using traditional medicine.’ (P12, 60-year-old female, Jan 2022)

This clarifies that the participant was referring to the communicative process through which relational openness encourages disclosure.

#### Managing hesitation

Participants described strategies for addressing patient hesitation by linking disclosure to treatment safety. P9 stated:

‘I ask the patient to tell me the truth, and when I discover they are using traditional medicine, I don’t dismiss them; instead, I speak appropriately. I show them that traditional medicines in this condition are not to be used because they will harm their kidneys.’ (P9, 51-year-old male, Jan 2022)

Participants added that trust could also be strengthened through gradual dialogue rather than direct questioning. P4 explained:

‘Uh, yes, it [*the disclosure process*] will depend on whether I see that the traditional medication will worsen the outcome I want to achieve. I will explain to the patient why he must first complete my [*AM*] treatment.’ (P4, 58-year-old female, Jan 2022)

This demonstrates how physicians balance patient autonomy with clinical safety while respecting privacy rules.

### Boundary coordination: Managing disclosed information

This theme reflects CPM theory’s concept of boundary coordination, which occurs when private information becomes shared and must be co-managed. Participants described how they handled disclosures of TM use and how collaborative communication could support shared responsibility between physicians and traditional health practitioners (THPs).

#### Responses to disclosure

Many physicians described adopting a cooperative approach when patients revealed TM use. P9 expressed:

‘They [*THPs*] must learn from us, and we must learn from them for healthcare to be united. Healthcare is not just for Western-trained doctors; THPs are also a part of our healthcare system.’ (P9, 51-year-old male, Jan 2022)

This reflects boundary coordination through mutual recognition of shared healthcare roles.

Participants also described acknowledging TM to validate patients’ experiences. P7 explained:

‘Look, if you happen to be using traditional medicine, feel free to disclose to the practitioner and don’t hold back. If they know you [*the physician*] acknowledge the existence of such a thing [*TM*], they will open up.’ (P7, 45-year-old male, Jan 2022)

Such statements illustrate how affirming the legitimacy of TM helps sustain coordinated boundaries between patients and practitioners.

#### Collaboration with traditional health practitioners

Several participants supported institutional collaboration with THPs. P6 suggested:

‘The Department of Health must involve, regulate, and train traditional medicine practitioners while also training them on when to refer patients to clinics if they believe the patient is too sick for them to handle. THPs and clinics or hospitals must have open lines of communication because they [*THPs*] serve as patients’ first point of contact with traditional treatment.’ (P6, 40-year-old female, Jan 2022)

This clarifies that ‘treatment’ referred to TM, highlighting the need for structured inter-system communication.

#### Integration challenges

Participants acknowledged persistent challenges. P2 noted:

‘Usually, doctors just laugh. I’m not sure why, but I know doctors would dismiss traditional medicine because we aren’t trained in it. We don’t know how it works, and it’s difficult to say that this one can’t be used with a certain drug.’ (P2, 68-year-old male, Dec 2021)

This view underscores how limited biomedical knowledge of TM constrains boundary coordination.

### Boundary turbulence: Challenges in non-disclosure

This theme aligns with CPM theory’s concept of boundary turbulence, which occurs when expectations of privacy or disclosure are violated, leading to uncertainty or conflict. Participants reported emotional strain and clinical challenges when TM use was discovered late in the care process.

#### Impacts of non-disclosure

Physicians expressed concern about adverse interactions and compromised care. P3 stated:

‘There are concerns about adverse reactions … it would be safe for them [*patients*] to make us aware of the conditions they think we cannot treat. Tell us they did and took the traditional route.’ (P3, 29-year-old female, Dec 2021)

Similarly, P7 suggested that open collaboration could mitigate such risks:

‘Collaboration with whoever is administering traditional medicine could help figure out how THPs determine dosage and how it affects patient management in allopathic medicine.’ (P7, 45-year-old male, Jan 2022)

These excerpts indicate that undisclosed TM use disrupts information flow, affecting treatment safety.

#### Conflict resolution

Participants discussed ways to restore open communication after turbulence. P13 explained:

‘Patients should be aware of the impact of their treatment plans on their condition, as well as how these interact with other treatments they may be using. If they know such information, it will be easier for them to disclose.’ (P13, 28-year-old male, July 2022)

This highlights education as a stabilising strategy to reduce turbulence.

#### Emotional and professional strain

Some participants described frustration when discovering undisclosed TM use. P10 shared:

‘When I discover that my patient is using traditional medicine, I cannot do anything as a doctor.’ (P10, 61-year-old female, Jan 2022)

To manage this tension, participants described maintaining empathy and re-establishing trust. P11 reflected:

‘Try to understand their [*patients’*] point of view and where they’re coming from. If they believe in traditional medicine, give them guiding advice and all the side effects of whatever they are taking, but never discourage them.’ (P11, 50-year-old female, Jan 2022)

These practices represent attempts to restore disrupted boundaries through supportive, non-judgemental communication.

## Discussion

This qualitative study examined physicians’ perspectives on communication dynamics surrounding TM disclosure in South Africa’s pluralistic healthcare landscape, using CPM theory as the guiding framework. Rather than exploring ‘how’ disclosure occurs through direct observation, the study explored physicians’ perceptions of the conditions, barriers and facilitators that influence TM disclosure within AM settings. The findings illuminate how privacy boundaries are negotiated through relational trust, cultural legitimacy and professional attitudes.

Communication privacy management theory proved analytically valuable for understanding how South African healthcare professionals interpret privacy and disclosure in intercultural encounters. Although developed in Western interpersonal contexts, CPM theory’s constructs of ownership, privacy rules, boundary coordination and boundary turbulence aligned closely with physicians’ accounts of TM communication. This integration demonstrates that CPM theory can be effectively contextualised within multicultural and dual-health-system environments, where disclosure is influenced by both interpersonal and systemic forces.

Physicians described patients’ control over TM information as an assertion of ownership, reflecting CPM theory’s first component. Many perceived that patients deliberately decide whether to reveal TM use based on anticipated judgement, stigma, or perceived relevance to AM care. These privacy rules are shaped by broader sociocultural norms and institutional hierarchies that privilege AM knowledge systems.^36,37^ This finding is consistent with previous South African and African research reporting that fear of disapproval or mockery discourages TM disclosure.^38^ While earlier studies conceptualised this reluctance as a communication barrier, the CPM theory perspective clarifies that withholding information is also a rational privacy-management strategy. By situating privacy ownership within a pluralistic context, the study expands understanding of how patients regulate sensitive health information in environments of unequal epistemic authority. Alternative explanations should also be considered. Patients’ non-disclosure of TM may not only originate from physicians’ attitudes but also reflect intentional privacy management and broader systemic influences, such as patients’ desire for cultural autonomy.^39^ Thus, non-disclosure can represent a contextual rational strategy shaped by power relations within South Africa’s pluralistic healthcare system.^40^

Trust emerged as central to effective boundary coordination between patients and physicians. Participants described how patients disclosed more readily when the consultation environment was conversational, respectful and non-judgemental. This supports international findings that relational trust can moderate disclosure of culturally sensitive information.^18^ Instead of cultural competence, the study underscores the importance of cultural safety and cultural humility, which prioritise physicians’ self-reflection and respect for patients’ lived experiences rather than prescriptive knowledge of cultural categories. Cultural safety recognises power imbalances in healthcare and seeks to create spaces where patients’ health perspective is validated,^21,41^ aligning with CPM theory’s emphasis on co-ownership of private information once disclosure occurs.^19^ When physicians acknowledged TM use without dismissal, they enabled coordinated management of shared information, reinforcing mutual respect and reducing boundary turbulence.

Boundary turbulence arose when physicians dismissed or undervalued TM, leading to patient withdrawal and non-disclosure. Participants noted that uncertainty about the actual TM used by patients and institutional policies often hindered open communication. This reflects the tension between allopathic expectations and patients’ holistic care practices. Consistent with CPM theory, turbulence occurred when privacy expectations were violated^42^ either by disregard for TM beliefs and attitudes regarding TM between patients and practitioners.^43^ Similar disruptions have been reported in a study from Ghana, where the health system operates a parallel model, with traditional and allopathic practitioners working separately rather than through coordinated, integrated care.^44^ Addressing turbulence, therefore, requires organisational and educational reforms that promote culturally safe dialogue and institutional recognition of TM within the broader healthcare framework.

This study illustrates how CPM theory can be adapted to analyse communication across plural medical systems. The findings demonstrate that disclosure of TM is neither a purely individual act nor a linear process; it is a socially negotiated practice situated within historical, cultural and institutional boundaries. South African physicians operate in a uniquely diverse health system where biomedical, traditional and spiritual practices intersect. Applying CPM theory in this setting broadens the theory’s relevance by showing how structural inequality and cultural stigma interact with privacy regulation.

Practically, these findings emphasise the need for culturally safe and dialogical communication strategies rather than cultural competence models. Training in cultural humility and relational communication could help physicians recognise their own positionality, respect patients’ explanatory models and engage in shared decision-making. Structured disclosure frameworks and cooperative platforms between TM and AM practitioners could further strengthen boundary coordination. Such initiatives might include joint workshops, formalised referral pathways and community education encouraging safe, voluntary disclosure. Legal and institutional recognition of THPs could also reduce patient secrecy and enhance integrative care. These implications extend CPM theory into applied health-communication practice, highlighting that privacy management is both relational and systemic.

### Theoretical and contextual contribution

Theoretically, this study extends CPM theory to an intercultural, interprofessional environment, demonstrating its applicability beyond Western interpersonal contexts. It reveals how ownership, privacy rules and boundary turbulence are shaped by the intersection of cultural identity, medical authority and systemic inequality. While, contextually, the study contributes to South African scholarship by illuminating how plural health perspectives complicate disclosure practices, it reframes TM disclosure not as a communication failure but as a contextually rational privacy strategy negotiated under conditions of power asymmetry. By focusing on physicians’ perspectives, the study offers an entry point into understanding institutional attitudes that shape patients’ disclosure experiences, a foundation for future research that could observe real-time disclosure interactions.

### Limitations

This study relied on self-reported perceptions of physicians rather than direct observation of clinical interactions. Consequently, the findings reflect physicians’ interpretations of disclosure processes rather than the processes themselves. Future research using observational or mixed-method approaches could more precisely capture how disclosure is co-constructed in practice.

The sample, drawn from four district hospitals in Gauteng, may not represent all South African physicians or other healthcare professionals, such as nurses and THPs. Furthermore, as interviews were conducted in English, some nuances of meaning may have been constrained. Despite these limitations, the findings provide valuable insights into physicians’ communicative positioning within South Africa’s dual healthcare system.

### Future research directions

Future research should extend this work by examining TM disclosure ‘in action’ through direct observation, video-recorded consultations or mixed-method approaches to capture the micro-interactional dynamics of privacy negotiation. Comparative studies involving patients, THPs and other healthcare workers could provide a more holistic understanding of how disclosure unfolds across professional and cultural boundaries. Longitudinal studies might also enable investigation of communication strategies that foster cultural safety and trust between TM and AM practitioners.

### Implications for policy and practice

The disclosure of TM use is a culturally mediated, privacy-regulated process influenced by relational trust and systemic legitimacy. Policies should promote cultural safety and humility training in medical curricula and continuing professional development to enhance ethical, inclusive communication. Establishing collaborative frameworks between TM and AM practitioners through mutual referral systems, shared learning initiatives and transparent disclosure protocols could strengthen coordination and reduce risks associated with non-disclosure. Public health initiatives should empower patients to discuss TM use confidently, reinforcing respect, shared responsibility and equitable healthcare delivery.

## Conclusion

This study offers a contextualised application of CPM theory in exploring physicians’ perceptions of TM disclosure within South Africa’s dual healthcare system. It highlights that disclosure is not merely a matter of patient willingness but a complex, relational negotiation influenced by cultural legitimacy, institutional trust and communication dynamics. By framing TM disclosure as a co-managed process between patients and physicians, the study extends CPM theory beyond Western interpersonal contexts into multicultural, pluralistic healthcare environments.

The findings demonstrate that physicians’ attitudes and communicative approaches can either support boundary coordination or trigger boundary turbulence, with direct implications for patient trust, safety and the quality of care. This study encourages a shift in focus from viewing non-disclosure as patient reluctance to examining the relational, institutional and cultural structures that shape disclosure opportunities within AM settings.
